# SAPS 3 as a predictor admission of surgical patients in the ICU

**DOI:** 10.1186/cc12630

**Published:** 2013-06-19

**Authors:** AMR Rosa de Oliveira, JM Silva, H Rocha, LM Sá Malbouisson, MJC Carmona

**Affiliations:** 1 Hospital das Clínicas da Faculdade de Medicina da Universidade de São Paulo, Cerqueira Cesar, São Paulo, SP, Brazil

## Introduction

Owing to lack of intensive care beds, patients undergoing intermediate-risk surgery are usually sent to the ward postoperatively. However, a part from this population evolves with complications requiring intensive care (ICU). The aim of the study was to evaluate the characteristics of surgical patients who were admitted to the ICU lately and to find predictors of the need for intensive care.

## Methods

**Figure 1:**
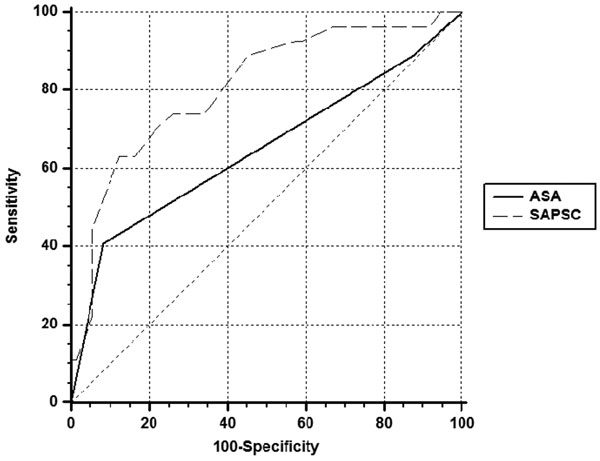


A prospective cohort study was performed in a tertiary hospital for 1 year. The study included patients with preoperative indication for ICU, but who at the end of surgery were taken to the ward postoperatively because of good clinical surgery. We evaluated the need for the ICU in this group, the SAPS 3 score preoperatively, the ASA physical status, demographics, origin and service requestor, need for blood transfusions intraoperatively, surgery time and hospital mortality. Patients undergoing palliative surgery were excluded. Independent predictors of the need for intensive care were assessed using logistic regression, with the sensitivity and specificity studied by ROC curve. See Figure 1.

## Results

We included 100 patients aged 66.4 ± 14.7 years. The SAPS 3 score average was 38.5 ± 8.6, 71% had ASA 2, women constituted 66% of casuistic. Most surgery was elective, the most frequent gynecologic (30%) and orthopedic (28%) and neuraxial regional anesthesia (49%). Of all patients, 27% required ICU admission, average on the sixth day after surgery and 3.0% died. The SAPS 3 score average was higher (45.4 ± 7.8 vs. 35.9 ± 7.4, *P *<0.001) and ASA 3 was more prevalent (40.7% vs. 8.2%, *P *= 0.001) in patients who required intensive care postoperatively. Furthermore, these patients had longer duration of surgery (4.2 ± 1.9 vs. 2.7 ± 1.5 hours, *P *<0.001), higher prevalence of gastrointestinal surgery (14.8% vs. 5.5%, *P *= 0.03) and greater need for intraoperative transfusion (18.5% vs. 5.5%, *P *= 0.04). In these patients admitted to the ICU mortality was 11.1% versus 0.0%, P = 0.004. In multivariate analysis, we found the value of SAPS 3 as an independent factor in determining whether the patient would need the ICU, OR = 1.25; 95% CI = 1.1 to 1.4; *P *= 0.001, and even the time of surgery, OR = 3.33; 95% CI = 1.7 to 6.3; *P *= 0.002. The ROC curve was 0.87; 95% CI = 0.78 to 0.93 for the SAPS 3 discriminating need for intensive care, rather than ASA, ROC 0.64; 95% CI = 0.54 to 0.74.

## Conclusion

The identification of high-risk surgical patients is a difficult task, but essential for their proper treatment, surgery time together with the SAPS 3 seem to be useful tools in this differentiation and may help to better characterize this population.

